# Supplementary material for: Specific lifestyle factors and in vitro fertilization outcomes in Romanian women: a pilot study
Source: PeerJ. 2022 Oct 4;10:e14189. doi: 10.7717/peerj.14189 (PMC9541609; doi:10.7717/peerj.14189)
Supplement: Supplemental Information 1 — in italic bold p < 0.05. Note: Poisson regression with robust error variance models with 192 degrees of freedom used to estimate relative risk (95% CI) for IVF outcomes in relation to women’s lifestyle habits and behaviours; aself-reported level of psychological stress (including work-related stress) on a level of 1 (low) to 3 (high); btotal number of grade 1 (best) and grade 2 (good) quality embryos based on the Istanbul consensus scoring system (Balaban et al., 2011) [file peerj-10-14189-s001.docx]

| Relative Risk (95% CI) | | | | | | | | | |
| --- | --- | --- | --- | --- | --- | --- | --- | --- | --- |
| Outcomes | Years spent smoking in the past | Years of exposure to passive smoke | Stress  level ^a^ | Routine weekly  exercise | Workout  duration (hours/per episode) | Monthly canned  food/beverage consumption | Monthly  fish consumption | Weekly  vegetable consumption | Weekly  fruit consumption |
| Oocyte fertilization | 1.02 (0.98, 1.05)  *p-value = 0.33* | 1.00 (0.98, 1.02)  *p-value =0.67* | 0.92 (0.71, 1.19)  *p-value = 0.51* | 0.99 (0.89, 1.10)  *p-value = 0.88* | 0.99 (0.70, 1.39)  *p-value = 0.96* | 1.03 (0.86, 1.23)  *p-value = 0.91* | 1.03 (0.85, 1.25)  *p-value = 0.79* | 1.12 (0.79, 1.59)  *p-value = 0.54* | 0.99 (0.82, 1.20)  *p-value = 0.92* |
| Embryo quality ^b^ | 1.03 (0.99, 1.07)  *p-value = 0.15* | 1.00 (0.97, 1.02)  *p-value = 0.83* | 1.00 (0.71, 1.39)  *p-value = 0.98* | 1.01 (0.88, 1.16)  *p-value = 0.91* | 1.13 (0.74, 1.71)  *p-value = 0.98* | 1.05 (0.83, 1.32)  *p-value = 0.61* | 1.16 (0.90, 1.48)  *p-value = 0.39* | 1.01 (0.64, 1.59)  *p-value = 0.98* | 0.96 (0.75, 1.23)  *p-value = 0.76* |
| Pregnancy | 1.08 (1.04, 1.13)  ***p-value < 0.001*** | 1.01 (0.98, 1.03)  *p-value = 0.65* | 0.37 (0.23, 0.59)  ***p-value < 0.001*** | 0.98 (0.85, 1.13)  *p-value = 0.57* | 1.04 (0.65, 1.65)  *p-value = 0.56* | 1.59 (1.27, 1.99)  ***p-value < 0.001*** | 0.98 (0.74, 1.29)  *p-value = 0.62* | 1.35 (0.81, 2.23)  *p-value = 0.25* | 1.40 (1.00, 1.97)  *p-value = 0.05* |
| Live birth | 1.12 (1.08, 1.18)  ***p-value < 0.001*** | 1.03 (1.00, 1.06)  *p-value = 0.07* | 0.26 (0.14, 0.48)  ***p-value < 0.001*** | 0.84 (0.69, 1.01)  *p-value = 0.54* | 0.51 (0.23, 1.13)  *p-value = 0.28* | 2.05 (1.57, 2.68)  ***p-value < 0.001*** | 1.13 (0.82, 1.55)  *p-value = 0.41* | 1.50 (0.80, 2.79)  *p-value = 0.20* | 1.53 (0.98, 2.36)  *p-value = 0.06* |

**Supplemental Table 1 (continued)**

| Relative Risk (95% CI) | | | | | | | | |
| --- | --- | --- | --- | --- | --- | --- | --- | --- |
| Outcomes | Weekly  use of face  cream | Weekly  use of cleansing  lotion | Weekly  use of body  lotion | Weekly  use of  perfume | Weekly  use of foundation  cream | Weekly use of lip and eyeliner | Weekly  use of  mascara | Weekly  use of  lipstick |
| Oocyte fertilization | 1.01 (0.92, 1.12)  *p-value = 0.81* | 1.00 (0.91, 1.10)  *p-value = 0.98* | 1.00 (0.90, 1.11)  *p-value = 0.99* | 0.98 (0.88, 1.08)  *p-value = 0.67* | 0.96 (0.86, 1.08)  *p-value = 0.49* | 0.98 (0.88, 1.09)  *p-value = 0.72* | 0.99 (0.90, 1.09)  *p-value = 0.86* | 0.97 (0.88, 1.07)  *p-value = 0.65* |
| Embryo quality ^b^ | 1.03 (0.91, 1.18)  *p-value = 0.63* | 1.04 (0.92, 1.17)  *p-value = 0.53* | 0.95 (0.92, 1.17)  *p-value = 0.41* | 0.97 (0.84, 1.10)  *p-value = 0.61* | 1.04 (0.90, 1.19)  *p-value = 0.60* | 1.08 (0.95, 1.23)  *p-value = 0.22* | 1.02 (0.90, 1.15)  *p-value = 0.79* | 0.96 (0.84, 1.09)  *p-value = 0.87* |
| Pregnancy | 0.73 (0.63, 0.83)  ***p-value < 0.001*** | 0.76 (0.65, 0.88)  ***p-value < 0.001*** | 0.76 (0.65, 0.88)  ***p-value = 0.01*** | 1.00 (0.87, 1.16)  *p-value = 0.98* | 0.79 (0.65, 0.96)  ***p-value = 0.02*** | 0.75 (0.61, 0.93)  ***p-value = 0.01*** | 0.95 (0.84, 1.08)  *p-value = 0.45* | 1.13 (0.99, 1.30)  *p-value = 0.06* |
| Live birth | 0.69 (0.58, 0.82) ***p-value < 0.001*** | 0.79 (0.66, 0.93)  ***p-value = 0.01*** | 0.79 (0.66, 0.93)  ***p-value = 0.01*** | 1.44 (1.15, 1.79)  ***p-value = 0.001*** | 0.73 (0.56, 0.95)  ***p-value = 0.02*** | 0.83 (0.66, 1.03)  *p-value = 0.09* | 1.21 (1.03, 1.43)  ***p-value = 0.02*** | 1.49 (1.23, 1.81)  ***p-value = 0.04*** |
