# Supplementary material for: Specific lifestyle factors and in vitro fertilization outcomes in Romanian women: a pilot study
Source: PeerJ. 2022 Oct 4;10:e14189. doi: 10.7717/peerj.14189 (PMC9541609; doi:10.7717/peerj.14189)
Supplement: Supplemental Information 2 — Variance explained (49%): 31% PCP-use; 18% healthy diet and exercise; *Factor loading score ≥ |0.50|. [file peerj-10-14189-s002.docx]

| Lifestyle habits and behaviours | PCP-use | Healthy diet and Exercise |
| --- | --- | --- |
| Level of stress | 0.30 | -0.15 |
| Weekly frequency of exercising | 0.16 | 0.69^*^ |
| Duration of each workout | 0.16 | 0.79^*^ |
| Weekly use of face cream | 0.59^*^ | 0.41 |
| Weekly use of cleansing lotion | 0.73^*^ | 0.15 |
| Weekly use of body lotion | 0.63^*^ | 0.08 |
| Weekly use of perfume | 0.80^*^ | -0.01 |
| Weekly use of foundation cream | 0.75^*^ | 0.09 |
| Weekly use of lip and eyeliner | 0.81^*^ | -0.05 |
| Weekly use of mascara | 0.87^*^ | -0.10 |
| Weekly use of lipstick | 0.49 | 0.19 |
| Monthly consumption of canned foods & beverages | -0.38 | -0.07 |
| Monthly consumption of fish | 0.17 | 0.49 |
| Weekly consumption of vegetables | -0.08 | 0.69^*^ |
| Weekly consumption of fruit | -0.29 | 0.83^*^ |
