# Supplementary material for: Specific lifestyle factors and in vitro fertilization outcomes in Romanian women: a pilot study
Source: PeerJ. 2022 Oct 4;10:e14189. doi: 10.7717/peerj.14189 (PMC9541609; doi:10.7717/peerj.14189)
Supplement: Supplemental Information 3 — in italic bold p < 0.05 Note: Poisson regression with robust error variance models with 187 degrees of freedom used to estimate relative risk (95% CI) for IVF outcomes in relation to women’s lifestyle patterns; avarimax rotated principal component describing women’s weekly use of several personal care products (face cream, face cleaning lotion, body lotion, perfume, foundation cream, lip and eyeliner, and mascara); bvarimax rotated principal component describing women’s weekly consumption of vegetables and fruit and their weekly frequency of exercise and duration of each workout; cn = 194 oocytes; dn = 79 embryos. [file peerj-10-14189-s003.docx]

|  | Relative Risk (95% CI) | | | |
| --- | --- | --- | --- | --- |
| *Outcomes* | *PCP-use ^a^* | *p-value* | *Healthy diet and physical activity ^b^* | *p-value* |
| Fertilized oocytes ^c^ | 1.00 (0.97, 1.03) | 0.75 | 1.00 (0.91, 1.09) | 0.95 |
| Embryo quality ^d^ | 1.01 (0.97, 1.05) | 0.67 | 1.00 (0.89, 1.13) | 0.94 |
| Pregnancy | 0.93 (0.89, 0.98) | ***0.003*** | 1.07 (0.94, 1.22) | 0.29 |
| Live birth | 0.99 (0.94, 1.04) | 0.66 | 0.96 (0.82, 1.13) | 0.64 |
