# Supplementary material for: Specific lifestyle factors and in vitro fertilization outcomes in Romanian women: a pilot study
Source: PeerJ. 2022 Oct 4;10:e14189. doi: 10.7717/peerj.14189 (PMC9541609; doi:10.7717/peerj.14189)
Supplement: Supplemental Information 4 — in italic bold p < 0.05. Note: Linear regression models with 188 degrees of freedom used to estimate mean difference (95% CI) for AMH, peak estradiol, and endometrial thickness as outcomes in relation to women’s lifestyle habits and behaviours; aself-reported level of psychological stress (including work-related stress) on a level of 1 (low) to 3 (high); bnegative binomial regression used to estimate the expected difference (95% CI) in antral follicle count as the outcome in relation to women’s lifestyle habits and behaviours ; AFC, antral follicle count; AMH, anti-Mullerian hormone. [file peerj-10-14189-s004.docx]

| Effect Estimate (95% CI) | | | | | | | | | |
| --- | --- | --- | --- | --- | --- | --- | --- | --- | --- |
| Outcomes | Years spent smoking in the past | Years of exposure to passive smoke | Stress  level ^a^ | Routine weekly  exercise | Workout  duration (hours/per episode) | Monthly canned  food/beverage consumption | Monthly  fish consumption | Weekly  vegetable consumption | Weekly  fruit consumption |
| Baseline AMH | -0.01 (-0.05, 0.04)  *p-value = 0.81* | -0.01 (-0.04, 0.01)  p-value = 0.30 | -0.14 (-0.51, 0.24)  *p-value = 0.47* | -0.03 (-0.14, 0.10)  *p-value = 0.59* | -0.10 (-0.52, 0.32)  *p-value = 0.87* | 0.14 (-0.10, 0.37)  *p-value = 0.52* | -0.27 (-0.55, 0.01)  *p-value = 0.43* | 0.55 (0.12, 0.97)  ***p-value = 0.01*** | 0.41 (0.14, 0.68)  ***p-value = 0.003*** |
| Baseline AFC ^b^ | 0.01 (0.003, 0.02)  ***p-value = 0.01*** | 0.02 (0.01, 0.02)  ***p-value < 0.001*** | -0.13 (-0.20, -0.06)  ***p-value < 0.001*** | -0.03 (-0.06, -0.01)  ***p-value =0.001*** | -0.05 (-0.8, -0.02)  ***p-value = 0.004*** | 0.14 (0.10, 0.19)  ***p-value < 0.001*** | -0.04 (-0.09, 0.02)  *p-value = 0.27* | -0.16 (-0.24, -0.08)  ***p-value < 0.001*** | 0.10 (0.05, 0.15)  ***p-value < 0.001*** |
| Endometrial thickness | 0.12 (0.08, 0.17)  ***p-value < 0.001*** | 0.03 (0.004, 0.05)  ***p-value = 0.02*** | -1.18 (-1.52, -0.85)  ***p-value < 0.001*** | -0.29 (-0.42, -0.17)  ***p-value = 0.01*** | -0.87 (-1.28, -0.47)  ***p-value = 0.001*** | 0.56 (0.34, 0.78)  ***p-value = 0.001*** | -0.43 (-0.71, -0.16)  ***p-value < 0.001*** | 0.16 (-0.28, 0.59)  *p-value = 0.47* | -0.09 (-0.36, 0.19)  *p-value = 0.52* |
| Peak estradiol | 12.0 (-21.0, 45.1)  *p-value = 0.47* | -8.15 (-25.0, 8.65)  *p-value = 0.34* | -677 (-910, -444)  ***p-value < 0.001*** | 151 (62.9, 238)  ***p-value = 0.01*** | 626 (356, 896)  ***p-value = 0.002*** | 82.3 (-75.5, 240)  *p-value = 0.93* | -74.2 (-263, 115)  *p-value = 0.06* | 335 (45.0, 624)  ***p-value = 0.02*** | 565 (399, 730)  ***p-value < 0.001*** |

**Supplemental Table 4 (continued)**

| Effect Estimate (95% CI) | | | | | | | | |
| --- | --- | --- | --- | --- | --- | --- | --- | --- |
| Outcomes | Weekly  use of face  cream | Weekly  use of cleansing  lotion | Weekly  use of body  lotion | Weekly  use of  perfume | Weekly  use of foundation  cream | Weekly use of lip and eyeliner | Weekly  use of  mascara | Weekly  use of  lipstick |
| Baseline AMH | -0.19 (-0.34, -0.04)  ***p-value = 0.01*** | -0.44 (-0.56, -0.33)  ***p-value < 0.001*** | 0.09 (-0.07, 0.25)  *p-value = 0.26* | 0.11 (-0.03, 0.26)  *p-value = 0.13* | -0.36 (-0.49, -0.23)  ***p-value < 0.001*** | -0.41 (-0.53, -0.29)  ***p-value < 0.001*** | -0.31 (-0.43, -0.19)  ***p-value < 0.001*** | 0.25 (0.13, 0.37)  ***p-value = 0.001*** |
| Baseline AFC ^b^ | -0.07 (-0.10, -0.04)  ***p-value < 0.001*** | -0.07 (-0.09, -0.04)  ***p-value < 0.001*** | 0.03 (0.003, 0.06)  ***p-value = 0.03*** | -0.04 (-0.07, -0.01)  ***p-value = 0.005*** | -0.06 (-0.09, -0.03)  ***p-value < 0.001*** | -0.07 (-0.09, -0.04)  ***p-value < 0.001*** | -0.05 (-0.08, -0.03)  ***p-value < 0.001*** | 0.02 (-0.01, 0.04)  *p-value = 0.05* |
| Endometrial thickness | -0.49 (-0.63, -0.36)  ***p-value < 0.001*** | -0.44 (-0.56, -0.33)  ***p-value < 0.001*** | 0.40 (0.24, 0.55)  ***p-value < 0.001*** | 0.06 (-0.09, 0.21)  *p-value = 0.42* | -0.19 (-0.33, -0.05)  ***p-value = 0.01*** | -0.11 (-0.24, 0.02)  *p-value = 0.11* | 0.09 (-0.03, 0.22)  p-value = 0.15 | 0.13 (0.002, 0.25)  ***p-value = 0.01*** |
| Peak estradiol | -33.2 (-137, 70.3)  *p-value = 0.53* | 43.3 (-45.3, 132)  *p-value = 0.34* | 78.1 (-30.0, 186)  *p-value = 0.16* | -66.2 (-165, 32.8)  *p-value = 0.19* | -96.3 (-189, -3.23)  ***p-value = 0.04*** | 2.61 (-86.6, 91.8)  *p-value = 0.95* | -126 (-211, -40.9)  ***p-value = 0.004*** | -28.9 (-113, 55.7)  *p-value = 0.30* |
