# Supplementary material for: Specific lifestyle factors and in vitro fertilization outcomes in Romanian women: a pilot study
Source: PeerJ. 2022 Oct 4;10:e14189. doi: 10.7717/peerj.14189 (PMC9541609; doi:10.7717/peerj.14189)
Supplement: Supplemental Information 5 — in italic bold p < 0.05. Note: Linear regression models with 192 degrees of freedom used to estimate mean difference (95% CI) for AMH, peak estradiol, and endometrial thickness as outcomes in relation to women’s lifestyle habits and behaviours; aself-reported level of psychological stress (including work-related stress) on a level of 1 (low) to 3 (high); bnegative binomial regression used to estimate the expected difference (95% CI) in antral follicle count as the outcome in relation to women’s lifestyle habits and behaviours ; AFC, antral follicle count; AMH, anti-Mullerian hormone. [file peerj-10-14189-s005.docx]

|  | Effect Estimate (95% CI) | | | | | | | | |
| --- | --- | --- | --- | --- | --- | --- | --- | --- | --- |
| Outcomes | Years spent smoking in the past | Years of exposure to passive smoke | Stress  level ^a^ | Routine weekly  exercise | Workout  duration (hours/per episode) | Monthly canned  food/beverage consumption | Monthly  fish consumption | Weekly  vegetable consumption | Weekly  fruit consumption |
| Baseline AMH | -0.05 (-0.10, -0.01)  ***p-value = 0.02*** | -0.02 (-0.05, 0.004)  *p-value = 0.09* | 0.01 (-0.33, 0.35)  *p-value = 0.93* | -0.13 (-0.26, 0.01)  *p-value = 0.56* | -0.11 (-0.56, 0.33)  *p-value = 0.13* | 0.14 (-0.10, 0.38)  *p-value = 0.56* | -0.48 (-0.73, -0.23)  ***p-value = 0.01*** | 0.43 (-0.02, 0.89)  *p-value = 0.06* | 0.08 (-0.17, 0.34)  *p-value = 0.53* |
| Baseline AFC ^b^ | -0.0004 (-0.01, 0.01)  *p-value = 0.94* | 0.01 (0.001, 0.01)  ***p-value = 0.01*** | 0.05 (-0.03, 0.13)  *p-value = 0.20* | -0.05 (-0.09, -0.01)  ***p-value = 0.01*** | -0.14 (-0.24, -0.03)  ***p-value = 0.02*** | 0.17 (0.12, 0.22)  ***p-value < 0.001*** | -0.15 (-0.21, -0.09)  ***p-value = 0.03*** | -0.18 (-0.29, -0.08)  ***p-value < 0.001*** | 0.01 (-0.05, 0.07)  *p-value = 0.83* |
| Endometrial thickness | 0.11 (0.07, 0.15)  ***p-value < 0.001*** | 0.04 (0.01, 0.06)  ***p-value = 0.003*** | -1.19 (-1.48, -0.89)  ***p-value < 0.001*** | -0.29 (-0.42, -0.16)  ***p-value = 0.04*** | -0.84 (-1.27, -0.41)  ***p-value = 0.004*** | 0.62 (0.40, 0.84)  ***p-value = 0.003*** | -0.46 (-0.71, -0.21)  ***p-value < 0.001*** | 0.08 (-0.37, 0.54)  *p-value = 0.71* | -0.06 (-0.31, 0.19)  *p-value = 0.65* |
| Peak estradiol | 22.1 (-9.06, 53.3)  *p-value = 0.16* | -7.52 (-24.7, 9.65)  *p-value = 0.39* | -488 (-707, -270)  ***p-value < 0.001*** | 143 (53.2, 232)  ***p-value = 0.01*** | 567 (276, 858)  ***p-value = 0.01*** | 203 (46.0, 360)  ***p-value = 0.02*** | -192 (-366, -17.9)  ***p-value = 0.002*** | 282 (-25.0, 589)  *p-value = 0.07* | 399 (238, 561)  ***p-value < 0.001*** |

**Supplemental Table 5 (continued)**

| Effect Estimate (95% CI) | | | | | | | | | |
| --- | --- | --- | --- | --- | --- | --- | --- | --- | --- |
| Outcomes | Weekly  use of face  cream | Weekly  use of cleansing  lotion | Weekly  use of body  lotion | Weekly  use of  perfume | Weekly  use of foundation  cream | Weekly use of lip and eyeliner | Weekly  use of  mascara | Weekly  use of  lipstick |  |
| Baseline  AMH | -0.22 (-0.34, -0.09)  ***p-value = 0.001*** | -0.35 (-0.46, -0.24)  ***p-value < 0.001*** | 0.13 (-0.01, 0.27)  *p-value = 0.06* | 0.04 (-0.10, 0.18)  *p-value = 0.57* | -0.32 (-0.46, -0.19)  ***p-value < 0.001*** | -0.43 (-0.55, -0.30)  ***p-value < 0.001*** | -0.26 (-0.38, -0.14)  ***p-value < 0.001*** | 0.21 (0.08, 0.33)  *p-value = 0.05* |  |
| Baseline AFC ^b^ | -0.12 (-0.14, -0.09)  ***p-value < 0.001*** | -0.11 (-0.13, -0.08)  ***p-value < 0.001*** | -0.03 (-0.06, 0.002)  *p-value = 0.06* | -0.08 (-0.11, -0.05)  ***p-value < 0.001*** | -0.05 (-0.08, -0.01)  ***p-value = 0.01*** | -0.07 (-0.10, -0.04)  ***p-value < 0.001*** | -0.07 (-0.10, -0.04)  ***p-value < 0.001*** | -0.02 (-0.05, -0.01)  ***p-value < 0.001*** |  |
| Endometrial thickness | -0.39 (-0.51, -0.27)  ***p-value < 0.001*** | -0.26 (-0.37, -0.15)  ***p-value < 0.001*** | 0.28 (0.15, 0.41)  ***p-value < 0.001*** | 0.12 (-0.02, 0.25)  *p-value = 0.09* | -0.20 (-0.34, -0.06)  ***p-value = 0.01*** | -0.07 (-0.21, 0.07)  *p-value = 0.33* | 0.14 (0.02, 0.26)  ***p-value = 0.03*** | 0.16 (0.04, 0.29)  *p-value = 0.76* |  |
| Peak estradiol | -140 (-226, -54.4)  ***p-value = 0.002*** | -14.6 (-94.9, 65.7)  *p-value = 0.72* | -27.6 (-120, 64.8)  *p-value = 0.56* | -70.9 (-163, 21.0)  *p-value = 0.13* | -85.4 (-182, 11.5)  *p-value = 0.08* | 24.9 (-69.2, 119)  *p-value = 0.60* | -98.9 (-181, -16.9)  ***p-value = 0.02*** | -33.1 (-120, 53.9)  *p-value = 0.12* |  |
