# Supplementary material for: Specific lifestyle factors and in vitro fertilization outcomes in Romanian women: a pilot study
Source: PeerJ. 2022 Oct 4;10:e14189. doi: 10.7717/peerj.14189 (PMC9541609; doi:10.7717/peerj.14189)
Supplement: Supplemental Information 6 — in italic bold p < 0.05. Note: Linear regression models with 192 degrees of freedom used to estimate mean difference (95% CI) for AMH, peak estradiol, and endometrial thickness in relation to women’s lifestyle patterns; avarimax rotated principal component describing women’s weekly use of several personal care products (face cream, face cleaning lotion, body lotion, perfume, foundation cream, lip and eyeliner, and mascara); bvarimax rotated principal component describing women’s weekly consumption of vegetables and fruit and their weekly frequency of exercise and duration of each workout; cnegative binomial regression used to estimate the expected difference (95% CI) in antral follicle count as the outcome in relation to women’s lifestyle patterns; n = 194 oocytes and n = 79 embryos; [file peerj-10-14189-s006.docx]

|  | Effect Estimate (95% CI) | | | |
| --- | --- | --- | --- | --- |
| *Outcomes* | *PCP-use ^a^* | *p-value* | *Healthy diet and physical activity ^b^* | *p-value* |
| Baseline AMH | -0.09 (-0.13, -0.06) | ***<0.001*** | -0.03 (-0.14, 0.09) | 0.64 |
| Baseline AFC ^c^ | -0.03 (-0.04, -0.03) | ***<0.001*** | -0.03 (-0.06, -0.01) | ***0.01*** |
| Endometrial thickness | -0.02 (-0.06, 0.02) | 0.33 | -0.21 (-0.33, -0.09) | ***<0.001*** |
| Peak estradiol | -28.7 (-52.9, -4.54) | ***0.02*** | 207 (130, 284) | **<*0.001*** |
