# Supplementary material for: Specific lifestyle factors and in vitro fertilization outcomes in Romanian women: a pilot study
Source: PeerJ. 2022 Oct 4;10:e14189. doi: 10.7717/peerj.14189 (PMC9541609; doi:10.7717/peerj.14189)
Supplement: Supplemental Information 8 [file peerj-10-14189-s008.doc]

***QUESTIONNAIRE***

We thank you for your willingness to participate in this study. You have been selected through a scientific procedure and your cooperation is very important for the success of this study. Please answer the questions as accurately as possible. All information collected in this study is confidential and will only be used for medical research.

**A. IDENTIFICATION**

UNIQUE IDENTIFICATION CODE (ID): DATE OF COMPLETION: ___________________

FIRST AND LAST NAME:_____________________________________________ Phone No._________________

(will not be entered into the database - identification will be done with a unique identification code)

ADDRESS WHERE YOU LIVE

CITY ____________________________STREET ______________________ No._____

**B. BACKGROUND**

B1. DATE OF BIRTH: _____ ____ ______ height___________(cm) weight____________(kg)

DAY MONTH YEAR

B2. What is the last graduate school? (**check the box with the correct answer)** □primary school (4 classes) □secondary school (8 classes) □vocational school □high school □post high school □college □faculty

**C. LIVING AREA, EXPOSURE AND OCCUPATIONAL EXPOSURE**

C1. For how many years have you lived in your current home? (**write in the box**) □□

C2. Is your house located in the vicinity of: (**mark the number next to the answer**) 1. a municipal / medical waste incinerator; 2. chemical industry; 3. tanneries; 4. metal coating workshop; 5. smelter; 6. high traffic roads?

C3. Are you employed? (**check the box with the correct answer**) □YES □NO

C4. Occupation______________________ C5. Workplace ______________________________

C6. For how many years have you been working (length of work)?________years

C7. Have you been exposed to toxic substances at work? **(check the box with the correct answer)** □YES □NO □I DON’T KNOW

**C8. If you answered YES to the previous question, please specify to what type of toxic substances have you been you exposed? (check the box in the table with the correct answer to the question and specify the exposure period as number of years, for each of the substances you checked in the table**)

| **Occupational exposure** | **No** | **Yes** | **Period you have been exposed (years)** |
| --- | --- | --- | --- |
| Arsenic-based pesticides |  |  |  |
| Mercury |  |  |  |
| Lead |  |  |  |
| Cadmium |  |  |  |
| Arsenic |  |  |  |
| Chromium |  |  |  |
| Other metals or substances (specify) |  |  |  |

**D. HEALTH STATUS**

D1. How old were you when you had your first period? (**write in the box your age**) □□ **years**

D2. Are your menstruations accompanied by abdominal pain? □YES □NO

D3. Are your periods regular? □YES □NO

D4. Specify the time period at which you have your period: 1. <25 days; 2. 26-33 days; 3. > 33 days; Duration of your period _________(days)

D5. Have you ever taken oral contraceptives? □YES □NO ; Specify for how long have you taken the pills ___________(years)

D6. Have you ever become pregnant without medical treatment? □YES □NO; Specify the time period for which you have tried to become pregnant __________(months)

D7. Have you ever had a spontaneous abortion or a loss? □YES □NO

D8. If YES, how many spontaneous abortions and how many losses you had?______ spontaneous abortions _____ losses

D9. Personal disease history: 1. endometriosis; 2. micropolycystic ovarian syndrome; 3. pelvic inflammatory disease

D10. Do you have diabetes and / or problems with glucose tolerance? □YES □NO

D11. Do you have / have had frequent skin allergy and/or skin rash? □YES □NO

D12. Do you have high blood pressure? □YES □NO

**E. LIFESTYLE**

E1. Do you smoke? □YES □NO

If you are a **SMOKER IN THE PRESENT**:

E2. How many cigarettes do you usually smoke in one day? _______no. of cigarettes

E3. For how long have you been smoking? _____ years

If you were a **SMOKER IN THE PAST**:

E4. How many cigarettes did you usually smoke per day? _______ no. of cigarettes

E5. How long did you smoke? ________years

E6. Since how many years have you quit smoking? ________years

E7. Are you / have been exposed to cigarette smoke generated by other people at home or at work, at least for 1 hour / day, at least one year? □YES □NO

E8. If you answered YES to the previous question, for how long have you been exposed to cigarette smoke generated by other people at home and/or at work? ________hours/day _________no. years

E9. How would you describe the level of stress in your life (including work-related stress)?: 1. high; 2. medium; 3. low.

E10. Do you exercise (including walking, running, etc.) at home or at the gym?□YES □NO; **Frequency**: 1. everyday; 2. 3-4 times/week; 3. 1-2 times/week; 4. < 1 once/week; 5. never; **Duration:** 1) 2 hours; 2) 1 hour; 3) < 1 hour.

E11. What cosmetics do you use ?: 1. face cream; 2. cleansing lotion; 3. body lotion; 4. chemical hair dye; 5. perfume (applied directly on the skin); 6. foundation cream; 7. eyeliner; 8. blush; 9. mascara; 10. lipstick; **Frequency of use**: 1. everyday; 2. 5-6 times/week; 3. 1-2 times/week; 4. <1 once/week; 5. never.

**Consumption of food and drink potentially contaminated with metals**

E12. Do you eat canned foods (metallic container) and/or canned beverages ((metallic container))? □YES □NO

E13. If YES, how often do you eat canned foods and beverages (from metal containers)? (**mark the number next to the answer**) 1. everyday; 2. 3-4 times/week; 3. once/week; 4. 2 times/month; 5. less than once/month.

E14. Do you eat ocean fish and/or fresh water fish? □YES □NO

E15. If YES, how often do you eat fish (including pastrami, fish sausages, etc.)? (**mark the number next to the answer**) 1. everyday; 2. 3-4 times/week; 3. once/week; 4. 2 times/month; 5. less than once/month.

E16. Do you eat raw and cooked vegetables in soups or other dishes (e.g. potatoes, carrots, onions, radishes, spinach, cauliflower, parsley etc.)? □YES □NO

E17. If YES, how often do you eat vegetables? (**mark the number next to the answer**) 1. everyday; 2. 3-4 times/week; 3. once/week.

E18. Do you eat raw fruit or fruit compote? □YES □NO

E19. If YES, how often do you eat fruits (including cakes with fruits)? (**mark the number next to the answer**) 1. everyday; 2. 3-4 times/week; 3. once/week; 4. less than once/week.

E20. From where do you buy the vegetables and fruits you eat? **(mark the number next to the answer**) 1. from the supermarket/groceries; 2. from your own garden; 3. from farms near your house; 4. the town market

**F. Partner information**

F1. What is your partner's age?_____________(years)

**Personal pathological history**

F2. Does your partner have diabetes and/or problems with glucose tolerance? □YES □NO

F3. Does your partner have high blood pressure? □YES □NO

**Thank you for collaboration.**
