# Supplementary material for: Specific lifestyle factors and in vitro fertilization outcomes in Romanian women: a pilot study
Source: PeerJ. 2022 Oct 4;10:e14189. doi: 10.7717/peerj.14189 (PMC9541609; doi:10.7717/peerj.14189)
Supplement: Supplemental Information 9 [file peerj-10-14189-s009.doc]

***CHESTIONAR***

Va multumim pentru bunavointa de a participa in acest studiu. Ati fost selectionat printr-o procedura stiintifica si cooperarea Dvs. este foarte importanta pentru succesul acestui studiu. Va rugam sa raspundeti la intrebari cat mai corect posibil. Toate informatiile obtinute prin intermediul acestui studiu sunt confidentiale si vor fi utilizate numai pentru cercetare medicala.

**A. IDENTIFICARE**

COD UNIC DE IDENTIFICARE (ID): DATA COMPLETARII: ___________________

NUME SI PRENUME:______________________________________________ Nr. telefon___________________

(nu vor fi introduse in baza de date – identificarea se va realiza cu un cod unic de identificare)

ADRESA UNDE LOCUITI

LOCALITATE ____________________________STRADA ______________________ Nr_____

**B. Date generale**

B1. DATA NASTERII: _____ ____ ______ inaltime___________(cm) greutate____________(kg)

ZI LUNA AN

B2. Care este ultima scoala absolvita? (**marcati cu un X in casuta raspunsul)** □scoala primara (4 clase) □gimnaziu (8 clase) □scoala profesionala □liceu □scoala postliceala □colegiu □facultate

**C. EXPUNERE IN ZONA DE LOCUIT si EXPUNERE OCUPATIONALA**

C1. De cand locuiti in locuinta curenta? (**scrieti in casuta alaturata numarul de ani**) □□

C2. Locuiti in vecinatatea (**incercuiti cifra din dreptul raspunsului**) 1. unui incinerator de deseuri municipale/medicale; 2. industie chimica; 3. atelier de tabacarie; 4. atelier de acoperiri metalice; 5. topitorie de metale; 6. artere de circulatie cu trafic intens

C3. Sunteti incadrata in munca? (**marcati cu un X in casuta raspunsul**) □DA □NU

C4. Ocupatie______________________ C5. Loc de munca ______________________________

C6. De cati ani lucrati sau cati ani ati lucrat (vechime in munca)?________ani

C7. Sunteti/ati fost expusa la substante toxice la locul de munca? **(marcati cu un X in casuta raspunsul)** □DA □NU □NU STIU

**C8. Daca ati raspuns cu DA la intrebarea anterioara, va rugam sa precizati la ce tip de substante toxice sunteti/ati fost expusa? (marcati cu un X in casuta din tabel corespunzatoare raspunsului Dvs. la intrebare si specificati** perioada expunerii ca numar de ani, pentru fiecare din substantele mentionate in tabel)

| **Expunere ocupationala** | **Nu** | **Da** | **Perioada cat ati fost expusa (ani)** |
| --- | --- | --- | --- |
| Pesticide pe baza de arsen |  |  |  |
| Mercur |  |  |  |
| Plumb |  |  |  |
| Cadmiu |  |  |  |
| Arsen |  |  |  |
| Crom |  |  |  |
| Alte metale sau substante (specificati) |  |  |  |

**D. STAREA DE SANATATE**

D1. Ce varsta aveati cand ati avut prima menstruatie? (**scrieti in casuta alaturata, varsta**) □□ **ani**

D2. Menstruatiile sunt dureroase? □DA □NU

D3. Ciclul menstrual este regulat? □DA □NU

D4. Precizati intervalul de timp la care apare menstruatia: 1. sub 25 zile; 2. 26-33; 3. peste 33 zile; Durata menstruatiei _________(zile)

D5. Ati luat vreodata contraceptive orale? □DA □NU ; Precizati cat timp le-ati luat _____________(ani)

D6. Ati ramas vreodata insarcinata fara sa faceti tratament? □DA □NU; Precizati durata de timp cat ati incercat pana sa obtineti aceasta sarcina __________(luni)

D7. Ati avut vreodata un avort spontan sau o sarcina oprita in evolutie? □DA □NU

D8. Daca DA, cate avorturi spontane si cate sarcini oprite in evolutie ati avut?______avorturi spontane _____ sarcini pierdute

D9. Antecedente personale patologice: 1. endometrioza; 2. sindrom de ovar micropolichistic; 3. boala inflamatorie pelvina

D10. Aveti diabet zaharat si/sau probleme de toleranta la glucoza? □DA □NU

D11. Aveti/ati avut in mod frecvent eritem (roseata) si/sau eruptii pe piele? □DA □NU

D12. Aveti hipertensiune arteriala? □DA □NU

**E. STIL DE VIATA**

E1. Fumati? □DA □NU

Daca sunteti **FUMATOARE IN PREZENT**:

E2. Cate tigari fumati in mod obisnuit, intr-o zi? _______nr. tigari

E3. De cat timp fumati? _____ ani

Daca **ati fost FUMATOARE**:

E4. Cate tigari ati fumat in mod obisnuit, intr-o zi? _______nr. tigari

E5. Cat timp ati fumat? ________ani

E6. De cat timp ati renuntat la fumat? ________ani

E7. Sunteti/ati fost expusa la fum de tigara generat de alte persoane acasa sau la serviciu, cel putin 1 ora/zi, cel putin un an? □DA □NU

E8. Daca ati raspuns cu DA la intrebarea anterioara, precizati de cat timp sunteti expusa/cat timp ati fost expusa la fum de tigara generat de alte persoane acasa si la serviciu? ________ore/zi _________nr. ani

E9. Cum ati caracteriza nivelul de stress din viata Dvs. (inclusiv stress-ul legat de serviciu)?: 1. ridicat; 2. mediu; 3. redus

E10. Faceti miscare (exercitii fizice, alergare, etc) acasa sau la sala de sport? □DA □NU; **Frecventa**: 1. zilnic; 2. 3-4 ori/saptamana; 3. 1-2 ori/saptamana; 4. < 1data/saptamana; 5. deloc; **Durata:** 1. 2 ore; 2. 1 ora; 3 < 1ora

E11. Ce produse cosmetice utilizati?: 1. creme de fata; 2. lotiune demachianta; 3. lotiuni pentru corp; 4. vopsea chimica pentru par; 5. parfum (aplicat direct pe piele); 6. fond de ten; 7. creion de ochi si buze; 8. fard de obraz; 9. rimel; 10. ruj de buze; **Frecventa de utilizare**: 1. zilnic; 2. 5-6 ori/saptamana; 3. 1-2 ori/saptamana; 4. <1 data/saptamana; 5. deloc

**Consum de alimente si bauturi potential contaminate cu metale**

E12. Consumati alimente din conserva (recipient metalic) si bauturi din doza ((recipient metalic)) ? □DA □NU

E13. Daca DA, cat de des consumati aceste alimente si bauturi din recipiente metalice? (**incercuiti cifra din dreptul raspunsului**) 1. zilnic; 2. de 3-4 ori/saptamana; 3. 1data/saptamana; 4. de 2 ori/luna; 5. mai putin de 1 data/luna

E14. Consumati peste oceanic si de apa dulce? □DA □NU

E15. Daca DA, cat de des consumati peste (inclusiv pastrama, carnati de peste etc)? (**incercuiti cifra din dreptul raspunsului**) 1. zilnic; 2. de 3- 4 ori/saptamana; 3. 1data/saptamana; 4. de 2 ori/luna; 5. mai putin de 1 data/luna

E16. Consumati legume crude si fierte in supe sau alte preparate (ex. cartofi, morcovi, ceapa, ridichi, spanac, conopida, patrunjel etc)? □DA □NU

E17. Daca DA, cat de des consumati legume? (**incercuiti cifra din dreptul raspunsului**) 1. zilnic; 2. de 3-4 ori/saptamana; 3. 1data/saptamana

E18. Consumati fructe crude sau in compot? □DA □NU

E19. Daca DA, cat de des consumati fructe (inclusiv din prajituri)? (**incercuiti cifra din dreptul raspunsului**) 1. zilnic; 2. de 4 ori/saptamana; 3. 1data/saptamana; 4. mai putin de 1 data/saptamana

E20. De unde provin legumele si fructele pe care le consumati? **(incercuiti cifra din dreptul raspunsului**) 1. de la supermarket/alte magazine de profil; 2. din gospodaria proprie; 3. de la ferme din vecinatatea locuintei; 4. piata din oras

**F. Informatii partener**

F1. Ce varsta are partenerul Dvs. de cuplu? _____________(ani)

**Antecedente personale patologice**

F2. Are partenerul Dvs. de cuplu diabet zaharat si/sau probleme de toleranta la glucoza? □DA □NU

F3. Are partenerul Dvs. de cuplu hipertensiune arteriala? □DA □NU

**Va multumim pentru colaborare.**
